# Atrial fibrillation in continuous flow-left ventricular assist device (CF-LVAD) patients: impact on pathophysiology

**DOI:** 10.1007/s10047-025-01532-9

**Published:** 2025-11-18

**Authors:** Nandini Nair, Dongping Du, Balakrishnan Mahesh

**Affiliations:** 1 Division of Cardiology, Department of Medicine, PSU College of Medicine, Hershey, PA USA; 2 Industrial, Manufacturing, Systems Engineering, Texas Tech University, Lubbock, TX USA; 3 Division of Cardiothoracic Surgery, Department of Surgery, PSU College of Medicine, Hershey, PA USA

**Keywords:** Atrial fibrillation, Post-LVAD implantation, CRT, LVAD, RV failure

## Abstract

Continuous Flow -Left Ventricular Assist Devices (CF-LVADs) remain the mainstay in end stage HFrEF patients with refractory Stage D HF. AF is common in patients supported on CF-LVADs, with a significantly increasing number developing new-onset AF post LVAD implantation. This review will summarize the effects of atrial fibrillation on LVAD physiology and its impact on outcomes. Literature search for this qualitative review was done using Google Scholar and PubMed. Only studies in adult human LVAD patients were included. The keywords used were atrial fibrillation in LVADs, effect of atrial fibrillation on functional mitral regurgitation /tricuspid regurgitation, effect on right ventricular failure, pump thrombosis and stroke and gender differences in outcomes.The main aspects addressed are effect on LVAD hemodynamics, the complexinteractions between low pulse pressure and atrial fibrillation leading to pump thrombosis and stroke , the role of atrial fibrillation in the initiation and progression ofright ventricular failure, impact on valvular regurgitation (Functional Mitral /TricuspidRegurgitation), utility of Cardiac Resynchronization Therapy post LVAD implant anduse of AI-driven technologies in risk factor identification to help identify novel risk factors important in predicting prognosis to improve outcomes. Use of AI technologies to identify novel risk factors for atrial fibrillation post LVADsurgery is warranted in the context of race, ethnicity and gender. Further studies areneeded to address optimal pacing strategies in LVAD patients with cardiac resynchronization therapy to institute guidelines for use of this therapy post LVAD implantation especially in the context of co-existing atrial fibrillation.

**Graphical abstract:**

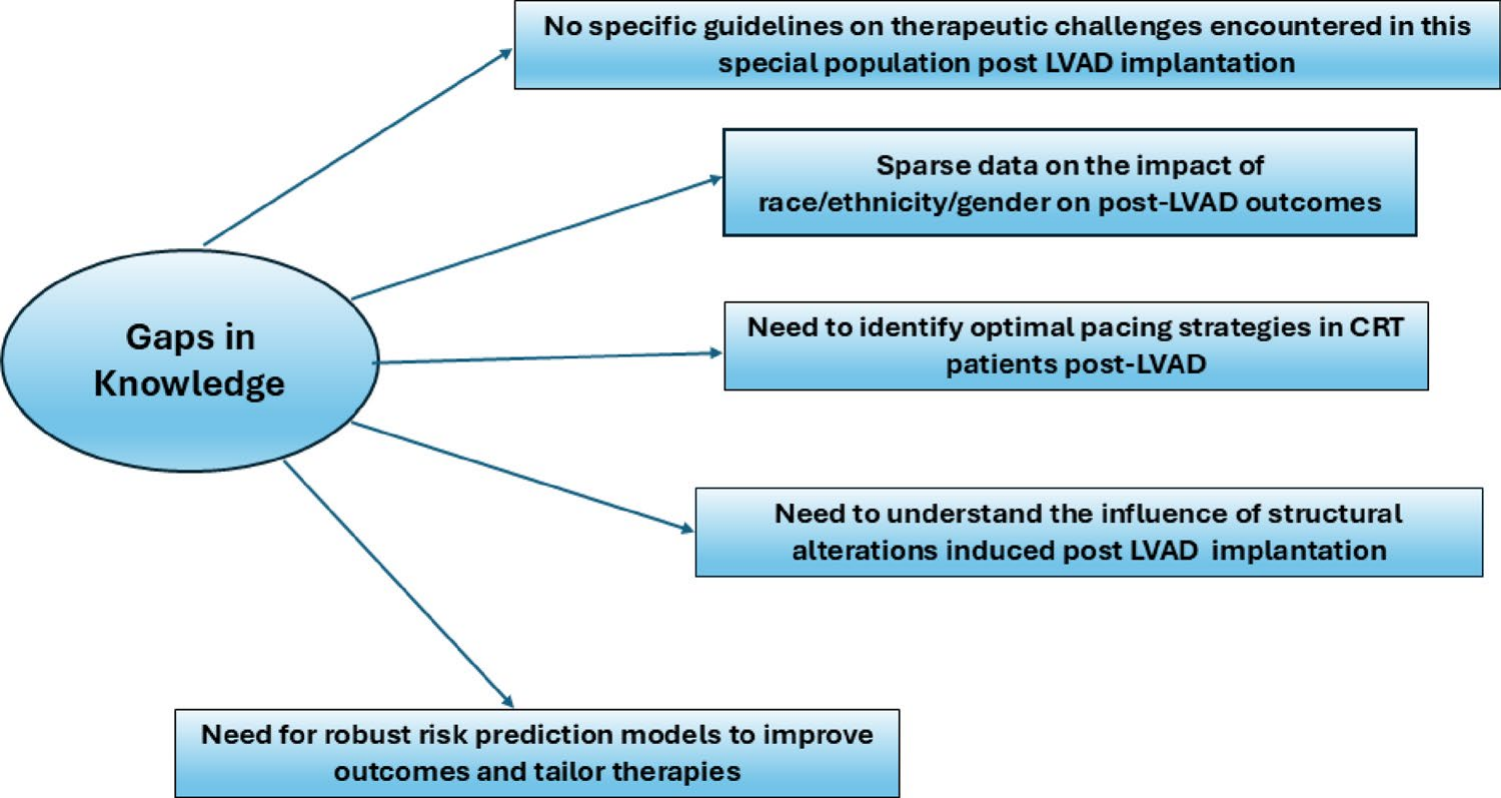

## Introduction

Left ventricular assist device (LVAD) therapy has emerged as standard of care for end stage heart failure. In the setting of donor heart shortage, the use of LVADs has increased. Atrial fibrillation (AF) remains common in this population. The incidence of AF in heart failure with reduced ejection fraction (HFrEF) patients in New York Heart Association (NYHA) class II–IV patients range from 15 to 50% and 20–50% in heart failure with preserved EF [1]. Prevalence of AF increases as the severity of heart failure (HF) increases [2]. AF and HF often coexist, with AF prevalence increasing with HF severity and age [2–4]. The annual incidence of new AF in HF patients can range from 2 to 5% and [3, 4].

The severe shortage of donor hearts and contraindications to heart transplant, makes it impossible for > 10% patients with advanced HF to benefit from transplant [5, 6]. LVAD use has therefore grown astronomically in the last two decades particularly with over 2500 implants in the United States and approximately 2000 LVADS implanted annually in Europe as a bridge to heart transplantation, decision, recovery or for life as destination therapy [7].

AF is common in patients supported on LVADs, with a significantly increasing number developing new-onset AF post LVAD implantation. Pre-existing AF is a major risk factor [8]. Incidence of new onset AF is reported to be about 8% after the initial 30 days of LVAD support [8–11]. In long term follow up the incidence of de-novo AF is estimated to be approximately 20–30% in LVAD patients. In LVAD patients with de-novo or recurrent paroxysmal AF, about 9% progress to persistent atrial fibrillation on long-term follow-up [9]. In about 15–25% patients with pre-LVAD AF there was resolution of AF post LVAD possibly secondary to favorable left atrial electrical remodeling [12]. New onset AF in the first year of LVAD support has been estimated to be about 10–20% [9, 12–14].

This review attempts to address the effect of AF in the LVAD population and the utility of risk factor identification for improving quality of life and outcomes. Data on the effect of AF on outcomes in this population would be valuable to better evaluate and treat LVAD patients to improve outcomes. The main aspects evaluated are effect of AF on LVAD hemodynamics, on mortality, on the complex interactions between low pulse pressure (PP), high blood pressure (BP) leading to pump thrombosis and stroke, impact on RV failure, association of Functional Mitral Regurgitation (FMR) /Functional Tricuspid Regurgitation (FTR) and AF, on use of intra cardiac defibrillators (ICDs) and Cardiac Resynchronization Therapy (CRT), the use of AI-driven technologies in risk factor identification for AF in the LVAD patients to improve outcomes and sex-based differences in the representation of women in the existing literature.

## Methods (Literature search)

**Fig. 1:**
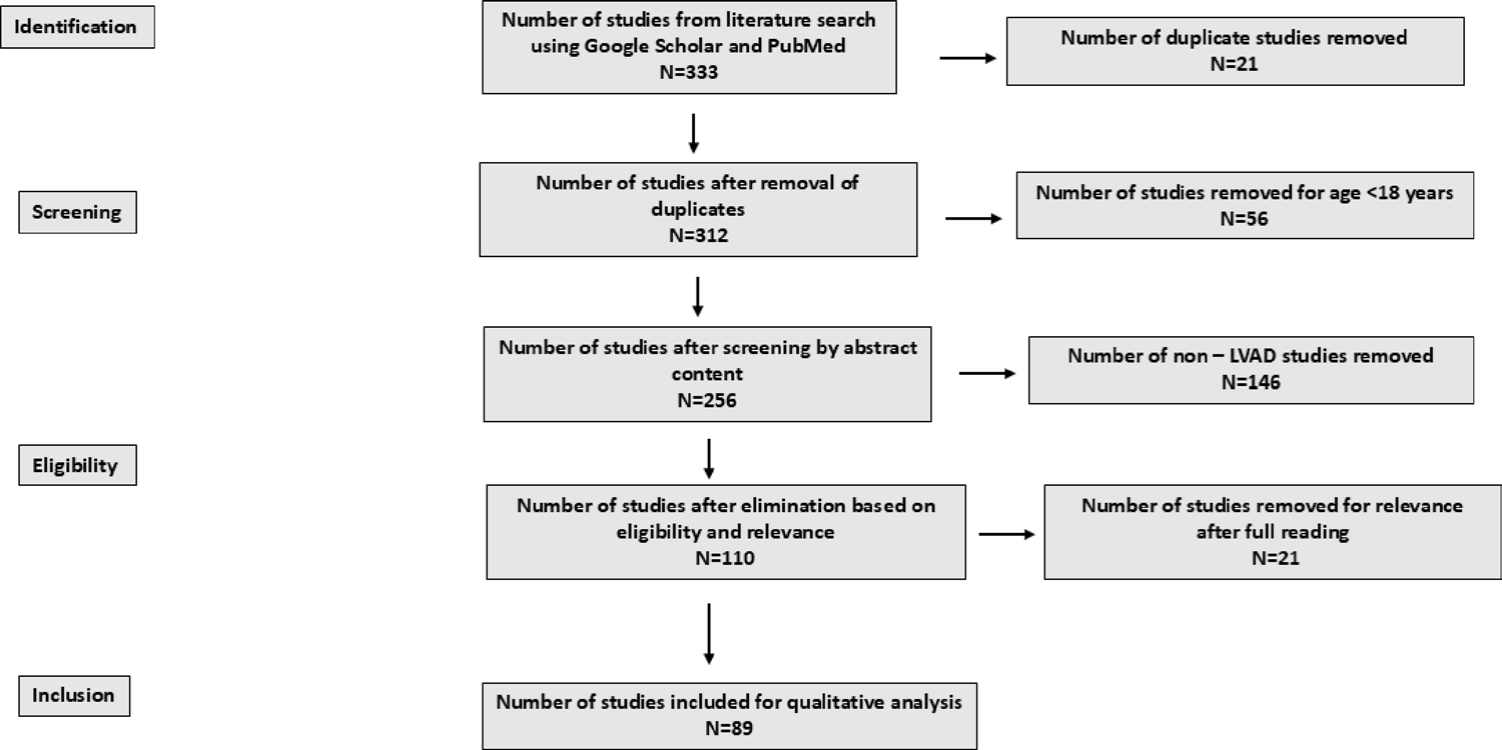
Details of literature search

The literature search for this qualitative review was done using Google Scholar and PubMed. This review included studies in the adult human population (> 18 years of age) who are supported on an LVAD. Figure 1 depicts details of the literature search conducted. A total of 333 studies were identified using Google scholar and PubMed searches. Of these 21 studies were noted as duplicates. After removing 56 papers for age < 18, 146 non -LVAD studies and 21 studies for relevance after full reading, 89 studies were included for qualitative analysis.

### Effect of AF on LVAD hemodynamics

The effect of AF on hemodynamics in LVAD-supported hearts should be considered pre and post implantation. Fast ventricular response to AF leads to progressive hemodynamic compromise of the right ventricle (RV) which in turn affects the systemic flow from the LVAD [12, 15]. It has been noted that elevated pulmonary capillary wedge pressures and right atrial pressure may predispose LVAD patients towards AF. Hence, AF episodes post implantation may signal suboptimal mechanical support and may serve as a noninvasive parameter for pump optimization [13]. Reverse electro-anatomical atrial remodeling leads to a reduction in the left atrial (LA) size and LA volume index [LAVi] [15]. Consequently, greater than 40% of patients with a history of AF would cease to have any recurrence in the long term [15]. Consistent with early observations of low blood flow velocities in the setting of increased spontaneous echo contrast and thromboembolism in non-LVAD patients it is not surprising that LVAD patients who undergo left atrial appendage occlusion have fewer thromboembolic events [16, 17]. On LVAD implantation there is a decrease in mitral regurgitation which can reduce atrial flow velocities and predispose to higher probability of clot formation therefore suggesting a benefit in occluding the left atrial appendage (LAA). LAA occlusion improved left atrial (LA) mechanical function by increasing LA reservoir. Such improvement was attributed to changes in loading conditions, suggesting a Frank-Starling mechanism rather than a change in the inherent contractility of the atrium [18]. About half of the LVAD patients show optimal central venous and wedge pressure at baseline speed settings [19]. LVAD patients in AF show augmented central venous pressure and lower LVAD flows [20, 21]. The consequent irregularity of the ventricular rate causes a slight decrease in cardiac output (approximately 6–7%), increase in right atrial pressure and pulmonary capillary wedge pressure [22, 23]. Though the systemic circulation is LVAD supported, arrhythmias can cause suboptimal LVAD flow and progressive RV failure. This hemodynamic impact of AF may be responsible for the different responses in LVAD flows when comparing LVAD patients with/without AF. The patients with LVAD and AF seem to have a higher incidence of aortic insufficiency than those without it though such a direct relationship is not well established in the current literature [24]. Larger studies are needed to assess the effect on post op hemodynamics. In LVAD patients, both Atrial Fibrillation (AF) and Aortic Insufficiency (AI) are significant complications, though the relationship is not a direct one-to-one cause-and-effect. AI is a common and often a progressive problem in LVAD patients, with higher incidence on the longer duration of support, potentially due to lack of aortic valve opening and subsequent leaflet changes. While AF is also prevalent in LVAD patients, it is more often a consequence of severe heart failure and structural heart remodeling. Managing both conditions requires complex, device-specific strategies to improve patient outcomes and avoid further implications [25].

### Effect of preoperative AF on mortality of LVAD patients

Table 1 shows a few studies addressing the common tenet of effect of preoperative AF on mortality. Tantrachoti et al. showed a significant effect of preoperative AF on mortality. This meta-analysis consisted of retrospective studies with risk of confounding factors, and severe comorbidities may have influenced the outcomes [22]. Kunihara et al. reported in a single center study that there was no significant effect of preoperative AF on mortality. In this study no differentiation was made between paroxysmal and persistent a fib which could have influenced the outcomes [23]. Usman et al. showed that preoperative AF was not associated with increased risk of all-cause mortality at 30 days. Discrepancies in the baseline characteristics and consequent heterogeneity in the clinical characteristics may have been a study limitation [26]. In a two-center retrospective analysis limited by patient heterogeneity preoperative AF did not impact late survival [27]. In a single center retrospective study Hui et al. showed that AF was a risk factor for ICU readmission in univariate analysis and that ICU admission was strongly associated with 1-year mortality in multivariable analysis (27). AF was not significantly associated with 90 day and 4 year mortality in a propensity-matched multivariate analysis by [28]. Prospective studies and precise identification of risk factors are needed to assess the exact role of preoperative AF on mortality.

**Table 1:** Selected studies on effect of preoperative atrial fibrillation on mortality of LVAD

Study	Study type	Number of subjects *N*	Duration of follow up	Conclusions
Tantrachoti et al. 2019	Meta-analysis	5823	7–24 months	Pre-operative AF may be associated with a higher mortality rate
Kurihara et al. 2018	Single center retrospective analysis	526	2000 days	No significant difference in post-operative stroke or survival
Usman et al. 2019	Meta-analysis	5,658	30 days	preoperative AF was not associated with increased risk of all-cause mortality at 30 days
Imamura et al. 2019	Japanese Mechanically Assisted Circulatory Support registry-retrospective analysis	190	1 year	No difference in survival free from Hemocompatibility-related adverse events (HRAEs)
Stulak et al. 2013	Retrospective analysis from 2 centers	389	2 years	Preoperative AF did not decrease late survival at 1 and 2 years after LVAD implant
Hui et al. 2019	Retrospective single center	287	1 year	ICU admission is strongly associated with 1 year mortality. with one of risk factors being preoperative atrial fibrillation
Antonides 2022	European Registry for Patients with Mechanical Circulatory Support	1821	Median follow up was 13.1 months	Preop atrial fibrillation was significantly associated with 90 day and 4 year mortality but this was not confirmed in the multivariable analysis

**Fig. 2:**
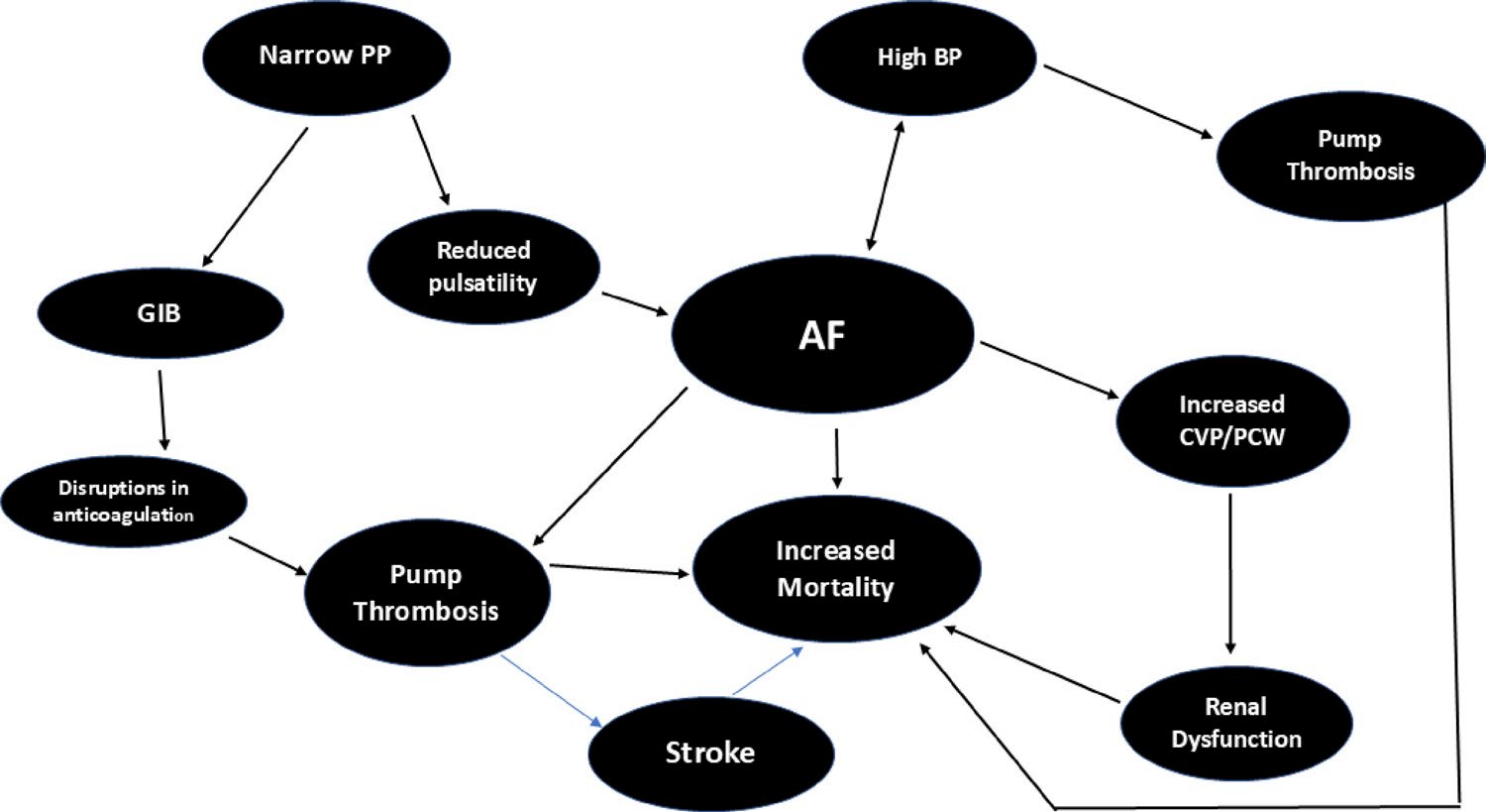
Complex interactions between AF, high BP, narrow PP in CF-LVADS

Figure 2 summarizes the complex interactions between BP, AF, Pump parameters/characteristics and pump thrombosis in the setting of the CF-LVAD. The narrow (PP) can lead to vascular/endothelial abnormalities which can in turn predispose to gastrointestinal bleeding (GIB) and reduced pulsatility leading to AF which in turn can increase mortality. AF also causes increased central venous pressure (CVP) /right atrial pressure (RAP) which leads to renal dysfunction and increased mortality. GIB can cause disruptions in anticoagulation predisposing to pump thrombosis which can in turn lead to embolic strokes and increased mortality.

## The complex interactions between low PP, high BP and AF in pump thrombosis and stroke

In patients with a continuous-flow left ventricular assist device (CF-LVAD), low pulse pressure and pulsatility are expected consequences of the device’s non-physiologic, constant blood flow. Atrial fibrillation (AF) significantly impacts patients with non-pulsatile continuous flow pumps, increasing the risk of adverse events and potentially worsening RV failure. While LVADs can stabilize hemodynamics even during ventricular fibrillation, the effects of AF on the right side of the heart can lead to serious complications. AF in CF-LVADs can increase heart failure readmission, stroke, and death. Persistent AF, in particular, is an independent predictor of increased mortality and hospitalizations for heart failure.

CF- LVADs support the left ventricle, but AF can disrupt the coordination of the right side of the heart. The loss of the atrial “kick” just before the ventricles contract reduces right ventricular (RV) output. This can lead to worsening right-sided heart failure symptoms, such as liver and venous congestion, even if the LVAD is functioning properly. Persistent AF is associated with a greater prevalence of significant tricuspid regurgitation, which further compromises RV function. The hemodynamics of the continuous flow pump can be influenced by arrhythmias. A reduction in the left ventricular preload, which can occur during AF, can decrease the overall flow through the device. AF is a known risk factor for blood clots and stroke. AF can exacerbate this by disrupting the heart’s native pump function, leading to a further reduction in pulsatility.

In the newer CF devices shear stress and stasis of blood appear to be lower as a consequence of the intrinsic pulsatility. The mechanism underlying the association between hypertension and pump thrombosis is possibly due to increased shearing forces of blood products as they pass through the pump. Hypertension is one of the major causes of AF. Pump thrombogenesis can be analogous to the Virchow’s triad in that the titanium pump surface acts as a nidus for the platelets to aggregate which is complemented by LVAD-induced hypercoagulability due to activated platelets followed by aberrant flow predisposing to thrombogenesis via shear stress-induced platelet activation (SIPA), hemolysis, and stasis. The genesis of a pump thrombus escalates the risk of a hemorrhagic event approximately 3 -fold most likely due to increased anticoagulation. The risk of thrombosis is greatest when mean arterial pressure (MAP) is > 90 mm Hg. Hence MAP < 80 mm Hg is considered optimal for BP control [27, 29–33].

The role of AF in pump thrombosis and adverse events is more complicated. In CF-LVAD patients, who already have a high risk of thromboembolism, AF can further compound this risk resulting in a higher incidence of stroke in LVAD patients with preoperative AF. Managing anticoagulation is critical and complex in LVAD patients with AF. They require careful monitoring to balance the risk of thromboembolic events with the increased risk of bleeding. Patients with existing implantable cardiac devices (pacemakers, defibrillators) need careful device management and programming after LVAD implantation, especially if AF develops. Such management strategies would likely prevent inappropriate shocks or device detection issues. However there are mixed results on long-term survival. Surgical interventions at the time of LVAD implantation for patients with pre-existing AF, such as LAA occlusion may be a better option [34]. Rhythm control with antiarrhythmic drugs or catheter ablation may be the option in worsening RV failure [35]. See figure 2 for complex interactions.

The newer pulsatile pumps in the pipeline that are hydraulically suspended need to be developed to suit the needs of individual patients. EVAHEART2 is a left ventricular assist device (EVA2) which has an improved design feature, and is further miniaturized, with lower pump speeds and an inflow cannula that does not protrude into the left ventricle. This pump may have the potential to mitigate the adverse outcomes of the CF-Flow pumps in the present day market. Augmenting arterial pulsatility can improve end-organ function and microcirculation by maintaining physiologic flow patterns, improving endothelial health and therefore improving patient outcomes [36].

Though AF is a risk factor for pump thrombosis it has been shown that many other factors come into play in this population though preventing clot formation via left atrial appendage could positively influence outcomes [36–38]. Larger studies with well-characterized populations are needed in this area for more definitive conclusions.

### Impact of AF on RV failure

The coexistence of HF and AF has been a longstanding point of discussion of cause and effect. In the sub cohort of the Framingham study it was noted that de novo AF was estimated at 37% in HF patients, and 57% of newly diagnosed HF patients had AF and lead to adverse events [34, 39]. Reduced systolic function of the left ventricle leads to structural and electrical remodeling of the atria, in response to increased left atrial filling pressures. Hence AF incidence increases as HF symptoms progress starting with 5% in NYHA class I to 50% in NYHA class IV [40–42]. Decompensated HF can worsen AF [39, 43] Hence in HF patients with LVADs the left ventricular filling remains unchanged by AF, but AF negatively impacts right ventricular filling leading to a compromise in cardiac output due to the loss of atrial contraction especially in patients with poor RV function and pulmonary hypertension [12]. In a small cohort of LVAD patients half of the population had AF with about a quarter having persistent AF, there was a significant associated with RV failure ( RVF) after adjusting for age [14]. It has also been noted that permanent AF patients tend to develop right ventricular failure earlier than those without AF predisposing to increased mortality in the LVAD population with AF [14]. In permanent preoperative-AF patients supported on HVAD or HM II, AF had no influence on survival rate, risk of stroke, pump thrombosis and gastrointestinal bleeding at the 1-year but at 2 year the AF cohort had a significant lower survival rate with much higher rate of RVF [44, 45]. Both AF (loss of atrial kick) and AV dyssynchrony (inefficient filling) can increase right atrial pressure. This elevation directly contributes to right-sided volume overload and congestion, a hallmark of RVF. The lack of atrial kick to support ventricular filling during AF leads to AV atrioventricular dyssynchrony, followed by septal deviation towards the left resulting in RV dilation and RVF. RV dilation can worsen tricuspid regurgitation and initiate RVF further compromising end organ function which can have a profound effect on LVAD supported patients. Persistent AF tends to be an independent predictor of mortality [44, 45]. These observations point to the role of AF influencing RVF in the CF centrifugal pumps like HVAD and the axial CF pumps like the HM II [45]. Further investigations are needed in the newer CF and pulsatile pumps. Hence these findings cannot be generalized to all LVADs.

### Functional valvular regurgitation and AF

 Functional Tricuspid Regurgitation (FTR) can occur as a consequence of AF in the LVAD population. In a single-center retrospective study of 133 patients who underwent HMIII implantation it was noted that patients with persistent AF had the worst survival. The primary end point of this study was death and a combined cardiovascular event consisting of death, stroke and hospital readmission at 1 year. It was also noted that the patients with residual TR at 1 month post LVAD implantation have a worse prognosis as compared to those without any residual TR. The etiology of FTR is mainly due RV dilation and dilation of the tricuspid annulus both of which occur as a consequence of AF due to left heart dysfunction. It is also important to note that LVAD implantation causes a distortion of the geometry of the RV especially at higher speeds in the HMIII population. In persistent AF patients an increase in tricuspid annulus diameter and increased severity of TR leads to augmentation of residual TR post LVAD implantation. Such changes were not seen in patients without AF and the presence of a LVAD. The shifting of the interventricular septum to the left could worsen the tricuspid annulus enlargement [46]. It is interesting that patients with permanent AF have poor outcomes in including mortality. However this trend is not noted in the patients with no AF or those with paroxysmal AF [46]. Further studies are needed to investigate this effect in defined populations.

 The concomitant presence of Functional Mitral Regurgitation (FMR) and persistent AF prior to LVAD implantation is a poor prognostic marker. LVAD implantation significantly alters mitral, left ventricular and left atrial geometry contributing to a reduction in LA size, LV size, and mitral annulus diameter. This leads to decreased residual MR post LVAD implantation. A single center retrospective study of 380 patients showed that the coexistence of FMR and AF in LVAD patients preimplantation considerably reduced survival at 2 years with a hazard ratio of 4.3 and was an independent marker of mortality [47]. This observation needs further validation in larger prospective studies with patient cohorts that are clearly demarcated as permanent AF, paroxysmal AF and those who have not had a diagnosis of AF.

### Association of AF with recurrent atrial and ventricular arrhythmias and the role of ICD/CRT post-LVAD

AF is common in HF patients with incidence increasing with severity of HF. In the perioperative period after LVAD implantation, many patients develop new-onset AF. In patients supported on LVADs, postoperative AF (POAF) is a common complication occurring in the first 30 days post implantation [9, 48]. POAF was seen in one study up to 28% in patients without a history of AF and the only risk predictor was COPD. This type of POAF did not have any impact on the mortality at 30 days but in the long term it had a significant association with ischemic stroke and pump thrombosis [10]. In another study patients without a prior history of AF had a 20% incidence of POAF which was associated with older age, moderate to severe MR, history of stroke and tricuspid surgery concomitantly. This cohort showed lot of resource utilization in terms of increasing renal failure, longer periods of ventilation and placement of unplanned RVADs [49].

Pre-existing AF is significantly associated with an increased risk of recurrent ventricular arrhythmias (VAs) after LVAD implantation. Suboptimal LVAD parameters can precipitate ventricular failure, further exacerbating the risk of VAs. Sustained VAs are fairly well tolerated in this population hence several observational studies do not show a significant survival benefit either with the use of ICD or CRT devices .

VAs can continue to manifest after LVAD implantation, most predominantly in the first 30 days after implantation especially in those LVAD patients who had a history of VAs before implantation [50–52]. It is unclear at this time if ICD improves survival post LVAD implantation as patients can continue to maintain cardiac output during rhythm disturbances [53].

The evidence on whether ICDs and CRT offer a survival advantage to patients with an LVAD has been mixed. Several single center and small observation studies have shown no statistical significance in survival benefit with ICDs [52–56] In a propensity matched study with INTERMACS data, the patients with an ICD showed an increased mortality risk and increased unexpected death when supported on a LVAD [55]. No significant increase in mortality was noted in a meta-analysis [57].

In CRT patients with LVAD, RV pacing appears to be better than CRT suggesting that the coronary sinus lead may be switched off for better results [58]. However, the exact benefits of CRT in LVAD patients is still unclear [25, 35, 58]. Further research is needed to understand the complex interaction of two devices namely the LVAD and the CRT in patients supported on both. To this effect interplay of factors should be studied possibly by Artificial Intelligence (AI)-driven technologies to unmask some of the yet to be discovered risk factors affecting the outcomes in these patients.

The right ventricular hemodynamics show higher CVP and pulmonary capillary wedge pressures compromising LVAD flows in patients in AF which in turn reduces cardiac output and increases the frequency of aortic regurgitation requiring intervention in AF patients. Additionally, AF can result in fast ventricular rates which can further compromise LVAD function and flow. In such cases controlling the rates and most importantly restoring sinus rhythm may be most influential in improving LVAD hemodynamics. However the management of AF patients on LVAD support has no universal guidelines due to knowledge gaps in identification of risk factors for AF in this population which may provide avenues and strategies to improve outcomes with efficient risk prediction.

In CF-LVAD patients with AF the management of CRT/ ICD is complex because AF can significantly hinder effective CRT pacing. While ICD therapy is generally recommended for sudden cardiac death prevention, guidelines suggest continued CRT-D management in LVAD patients, though the exact benefit is still being investigated. Patients with AF who have CRT-Ds may benefit from antiarrhythmic drugs, rate control, or AV nodal ablation to optimize biventricular pacing and improve outcomes, but this requires careful collaboration between LVAD and electrophysiology teams. Additionally the geometric change of the ventricles post lvad implantation may significantly impact the efficacy of CRT.

### Sex -based differences

Sex- based differences are difficult to pinpoint due to lack of solid evidence. In a large database study from the National Inpatient Sample(NIS) after adjustments for demographics and comorbidities were done, AF was associated with reduced thromboembolic events and in-hospital mortality in general [3]. Women seem to have fewer LVADs implanted, have a higher incidence of AF and also seem to experience a higher number of hemorrhagic strokes [59]. A trend towards higher number of strokes in females is noted despite the fact that women seem to be younger than their male counterparts and have fewer comorbidities. The mechanisms underlying this are not fully understood but sex-related hormonal influence on hemocompatibility, endothelial activation system and anticoagulation management have been grossly implicated [60]. Further studies are required to understand differences in stroke incidence in women with AF versus their male counterparts [60, 61].

Women with HF have always been found to be referred for advanced surgical therapies at tertiary and quaternary care centers providing advanced HF services at a consistently low rate [62, 63]. Gender disparities have been noted in registries where there is a much lower representation than in the real world [64–67]. It is unclear if this discrepancy is due to the later presentation of heart disease in women than men. Differences in inclusion in clinical trials, socio-economic differences, cultural or psychological issues leading to poor judgement of HF signs and symptoms cannot be excluded in this trend and may have further aggravated the underrepresentation of female patients. Such gender imbalance in registries and studies can skew data about gender-specific differences. Therefore, more studies are needed to define sex- based differences in outcomes.

**Fig. 3:**
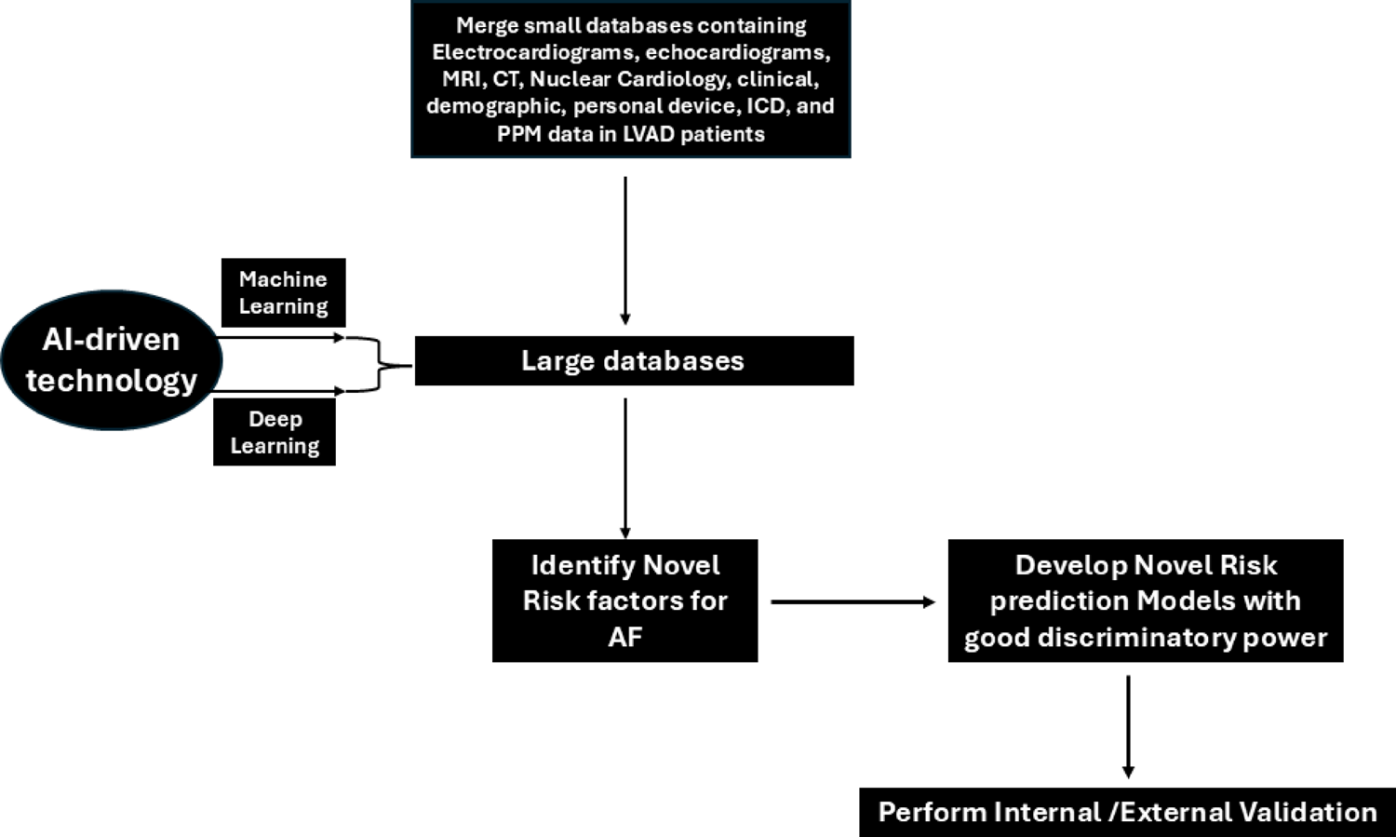
AI-driven approach to risk factor identificarion/prediction

Figure 3 shows the important aspects of collecting multimodal data to merge into a large database and the use of AI driven technologies to harness the data to identify risk factors and construct risk prediction models with better discriminatory power to improve patient outcomes.

### Use of AI-driven technologies in risk factor identification

Risk factor identification and risk prediction is still difficult in these complex interactions that occur in the LVAD population. Using the multimodal approach of analyzing multiple types of data collectively to produce a single large database allows for the construction of more robust databases than the data from a single modality. Different AI technologies can be used to extract risk factors from different modalities, which can then be fused for downstream prediction. For instance, convolutional neural networks for images such as ultrasound and computed tomography (CT), recurrent neural networks for time series like ECG and photoplethysmography, and gradient boosting models for tabular data such as demographics and lab results are some of the solid examples of use of AI in clinical applications to help improve outcomes. Large databases can be constructed by merging small databases containing electrocardiograms, echocardiograms, magnetic resonance imaging (MRI), and computed tomography (CT), clinical, demographic, personal and remote monitoring devices, data from intracardiac defibrillator (ICD), and permanent pacemaker (PPM) data in LVAD patients. Another imaging technique that can be used to incorporate data into large databases is single photon emission computed tomography (SPECT) which creates detailed three-dimensional images of organs and tissues. AI driven technologies such as machine learning and deep learning can be used to identify new risk factors from such large databases. The pooled risk factors can be used to develop robust novel risk prediction models with good discriminatory power. Finally, internal and external validation can be performed to test the universal applicability of the model. This may take many refinements in modelling to achieve fine definition. See figure 3.

A number of investigations are underway in using AI driven technologies for diagnosis of AF in patients not supported on LVADs. The use of electrocardiogram data in predicting AF has been investigated with robust discriminatory power. Studies using ECG with deep learning (DL) techniques show promise with improved c-statistic or discriminatory power [68–73]. A convolutional neural network was developed to detect the electrocardiographic signature of AF present during normal sinus rhythm at an c statistic of 0.87 [69]. The AI-ECG platform was applied to predict future AF, which had a c-statistic of 0.69 on its own and 0.72 combined with the CHARGE-AF clinical risk score [71]. Other methodologies used are photoplethysmography using wearable devices and smart phones [64–66]. The use of AI to predict success in cardioversion has been reported using machine learning (ML) [74–76]. Application of DL and neural networks to distinguish AF from other atrial arrhythmias have shown success [77–81]. ML and DL technology have been used to predict success of catheter ablations [85, 86] These investigations have been done in the non-LVAD populations and hence will need to be tested and validated in patients with LVADs. It will therefore involve considerable amount of future research to identify risk factors and develop risk prediction models in LVAD patients as there are challenges due to the presence of the LVAD itself in these patients.

### Strengths

This review highlights the impact of atrial fibrillation on the management of patients supported on CF-LVADs and the knowledge gaps that exist currently in this area of investigation.

### Limitations

This review is limited by the fact that most of the articles that were found in the current literature search were single center and/or retrospective studies.

### Future directions

Using AI technology, novel risk factors need to be identified to further understand the structural alterations and changes in the geometry introduced by LVAD implantation. Additionally, novel risk factor identification and risk prediction models are needed to improve targeted treatment strategies for different clinical scenarios. This is especially important in the context of lack of current knowledge of race, ethnicity and gender variations in LVAD patient outcomes. Tailoring the risk of individual patients through advances in technology forms the basis of personalized medicine. The existence of HF and AF in the setting of the LVAD is complex and needs to be defined and studied in more detail. The patient population is challenging due to the complex comorbidities that coexist [82].

The studies conducted in LVAD populations have limited definition of race and ethnicity to add another dimension to the challenges. The existing risk factors for AF in the LVAD population will need to be validated and used for further development and validation of risk prediction models. Current risk prediction models are essentially developed in the populations without LVADs hence it is difficult to assume that the same risk factors would affect the LVAD population especially the patients who develop AF de novo after LVAD implantation. Since AF can have multiple effects on patients supported on LVADs each population will need to be studied separately and the common denominators will need to be identified if possible to arrive at a generalized model.

## Conclusions

The existing risk factors will need to be tested and novel risk factors need to be identified in the context of race, ethnicity and gender. Female patient representation needs to be improved in studies to address gender based differences. Data on race and ethnicity variations are currently scanty or lacking. Use of AI driven technologies to identify novel risk factors may pave the way to better understand risk factors influencing outcomes in LVAD patients. Further studies are needed to address optimal pacing strategies in patients with CRT undergoing LVAD therapy. The role of alterations in geometry of the left atrium and ventricle in contributing to increased residual MR/TR post LVAD implantation needs further investigation. Knowledge gaps exist in the areas of sex-specific risk factors for prevalence of AF, differences in AF-associated stroke prognosis in the different sexes, differences in electrical and structural mechanisms in the two sexes and the role of hormonal differences in the different sexes.

## Data Availability

Data will be availble from the provided references as this is a review article.

## References

[1] Gopinathannair R, Chen LY, Chung MK, Cornwell WK, Furie KL, Lakkireddy DR, Marrouche NF, Natale A, Olshansky B, Joglar JA, American Heart Association Electrocardiography and Arrhythmias Committee and Heart Failure and Transplantation Committee of the Council on. Clinical Cardiology; Council on Arteriosclerosis, thrombosis and vascular Biology; Council on Hypertension; Council on lifestyle and cardiometabolic Health; and the stroke Council. Managing atrial fibrillation in patients with heart failure and reduced ejection fraction: a scientific statement from the American heart association. Circ Arrhythm Electrophysiol. 2021;14:HAE0000000000000078. 10.1161/HAE.0000000000000078. 34129347 10.1161/HAE.0000000000000078

[2] Maisel WH, Stevenson LW. Atrial fibrillation in heart failure: epidemiology, pathophysiology, and rationale for therapy. Am J Cardiol. 2003;91:2–8. 10.1016/s0002-9149(02)03373-8. 10.1016/s0002-9149(02)03373-8 12670636

[3] Gorter TM, van Veldhuisen DJ, Mulder BA, Artola Arita VA, van Empel VPM, Manintveld OC, Tieleman RG, Maass AH, Vernooy K, van Gelder IC, Rienstra M. Prevalence and incidence of atrial fibrillation in heart failure with mildly reduced or preserved ejection fraction: (Additive) value of implantable loop recorders. J Clin Med. 2023;12:3682. 10.3390/jcm12113682. 37297876 10.3390/jcm12113682 PMC10253837

[4] Chelikam N, Katapadi A, Venkata Pothineni N, Darden D, Kabra R, Gopinathannair R, Lakkireddy D. Epidemiology of atrial fibrillation in heart failure. Card Electrophysiol Clin. 2025;17(1):1–11. 10.1016/j.ccep.2024.08.004. 39893032 10.1016/j.ccep.2024.08.004

[5] Ponikowski P, Voors AA, Anker SD, Bueno H, Cleland JGF, Coats AJS, Falk V, González-Juanatey JR, Harjola VP, Jankowska EA, Jessup M, Linde C, Nihoyannopoulos P, Parissis JT, Pieske B, Riley JP, Rosano GMC, Ruilope LM, Ruschitzka F, Rutten FH, van der Meer P, ESC Scientific Document Group. 2016 ESC guidelines for the diagnosis and treatment of acute and chronic heart failure: the task force for the diagnosis and treatment of acute and chronic heart failure of the European society of cardiology (ESC)Developed with the special contribution of the heart failure association (HFA) of the ESC. Eur Heart J. 2016;37:2129–200. 10.1093/eurheartj/ehw128. 27206819 10.1093/eurheartj/ehw128

[6] Aissaoui N, Morshuis M, Maoulida H, Salem JE, Lebreton G, Brunn M, Chatellier G, Hagège A, Schoenbrodt M, Puymirat E, Latremouille C, Varnous S, Ouldamar S, Guillemain R, Diebold B, Guedeney P, Barreira M, Mutuon P, Guerot E, Paluszkiewicz L, Hakim-Meibodi K, Schulz U, Danchin N, Gummert J, Durand-Zaleski I, Leprince P, Fagon JY. Management of end-stage heart failure patients with or without ventricular assist device: an observational comparison of clinical and economic outcomes. Eur J Cardiothorac Surg. 2018;53(1):170–7. 10.1093/ejcts/ezx258. 28950304 10.1093/ejcts/ezx258

[7] Gustafsson F, Rogers JG. Left ventricular assist device therapy in advanced heart failure: patient selection and outcomes. Eur J Heart Fail. 2017;19:595–602. 10.1002/ejhf.779. 28198133 10.1002/ejhf.779

[8] Gopinathannair R, Cornwell WK, Dukes JW, Ellis CR, Hickey KT, Joglar JA, Pagani FD, Roukoz H, Slaughter MS, Patton KK. Device therapy and arrhythmia management in left ventricular assist device recipients: a scientific statement from the American heart Association. Circulation. 2019;139:e967–89. 10.1161/CIR.0000000000000673 30943783 10.1161/CIR.0000000000000673

[9] Deshmukh A, Bhatia A, Anyanwu E, Ota T, Jeevanandam V, Uriel N, Tung R, Ozcan C. Incidence and outcomes of postoperative atrial fibrillation after left ventricular assist device. ASAIO J. 2018;64:581–5. 10.1097/MAT.0000000000000763. 29485424 10.1097/MAT.0000000000000763 PMC7055419

[10] Hawkins RB, Mehaffey JH, Guo A, Charles EJ, Speir AM, Rich JB, Quader MA, Ailawadi G, Yarboro LT, Virginia Cardiac Services Quality Initiative. Postoperative atrial fibrillation is associated with increased morbidity and resource utilization after left ventricular assist device placement. J Thorac Cardiovasc Surg. 2018;156:1543–e15494. 10.1016/j.jtcvs.2018.03.169. 29801690 10.1016/j.jtcvs.2018.03.169 PMC6156995

[11] Enriquez AD, Calenda B, Gandhi PU, Nair AP, Anyanwu AC, Pinney SP. Clinical impact of atrial fibrillation in patients with the heartmate II left ventricular assist device. J Am Coll Cardiol. 2014;64:1883–90. 10.1016/j.jacc.2014.07.989. 25444141 10.1016/j.jacc.2014.07.989

[12] Noll AE, Adewumi J, Amuthan R, Gillombardo CB, Mannan Z, Kiehl EL, Hussein AA, Chung MK, Wazni OM, Starling RC, Soltesz EG, Cantillon DJ. Atrial tachyarrhythmias among patients with left ventricular assist devices: prevalence, clinical outcomes, and impact of rhythm control strategies. JACC Clin Electrophysiol. 2019;5:459–66. 10.1016/j.jacep.2018.11.016. 31000099 10.1016/j.jacep.2018.11.016

[13] Hickey KT, Garan H, Mancini DM, Colombo PC, Naka Y, Sciacca RR, Abrams MP, Solove M, Zeoli N, Flannery M, Garan AR, Biviano AB. Atrial fibrillation in patients with left ventricular assist devices: incidence, predictors, and clinical outcomes. JACC Clin Electrophysiol. 2016;2:793–8. 10.1016/j.jacep.2016.03.009. 29759763 10.1016/j.jacep.2016.03.009

[14] Kadado AJ, Akar JG, Hummel JP. Arrhythmias after left ventricular assist device implantation: incidence and management. Trends Cardiovasc Med. 2018;28:41–50. 10.1016/j.2017.07.002. 28734595 10.1016/j.tcm.2017.07.002

[15] Deshmukh A, Kim G, Burke M, Anyanwu E, Jeevanandam V, Uriel N, Tung R, Ozcan C. Atrial arrhythmias and electroanatomical remodeling in patients with left ventricular assist devices. J Am Heart Assoc. 2017;6:e005340. 10.1161/JAHA.116.005340. 28275069 10.1161/JAHA.116.005340 PMC5524037

[16] Fatkin D, Kelly RP, Feneley MP. Relations between left atrial appendage blood flow velocity, spontaneous echocardiographic contrast and thromboembolic risk in vivo. J Am Coll Cardiol. 1994;23:961–9. 10.1016/0735-1097(94)90644-0. 8106703 10.1016/0735-1097(94)90644-0

[17] Deshmukh A, Bhatia A, Sayer GT, Kim G, Raikhelkar J, Imamura T, Ozcan C, Ota T, Jeevanandam V, Uriel N. Left atrial appendage occlusion with left ventricular assist device decreases thromboembolic events. Ann Thorac Surg. 2019;107(4):1181–6. 10.1016/j.athoracsur.2018.09.004. 30365959 10.1016/j.athoracsur.2018.09.004

[18] Coisne A, Pilato R, Brigadeau F, Klug D, Marquie C, Souissi Z, Richardson M, Mouton S, Polge AS, Lancellotti P, Lacroix D, Montaigne D. Percutaneous left atrial appendage closure improves left atrial mechanical function through Frank-Starling mechanism. Heart Rhythm. 2017;14:710–6. 10.1016/j.hrthm.2017.01.042. 28188931 10.1016/j.hrthm.2017.01.042

[19] .Imamura T, Chung B, Nguyen A, Sayer G, Uriel N. Clinical implications of hemodynamic assessment during left ventricular assist device therapy. J Cardiol. 2018;71:352–8. 10.1016/j.jjcc.2017.12.001. 29287808 10.1016/j.jjcc.2017.12.001 PMC5811397

[20] Imamura T, Kinugawa K, Ono M, Kinoshita O, Fukushima N, Shiose A, Matsui Y, Yamazaki K, Saiki Y, Usui A, Niinami H, Matsumiya G, Arai H, Sawa Y. Implication of preoperative existence of atrial fibrillation on hemocompatibility-related adverse events during left ventricular assist device support. Circ J. 2019;83:1286–92. 10.1253/circj.CJ-18-1215. 31019163 10.1253/circj.CJ-18-1215

[21] Clark DM, Plumb VJ, Epstein AE, Kay GN. Hemodynamic effects of an irregular sequence of ventricular cycle lengths during atrial fibrillation. J Am Coll Cardiol. 1997;30:1039–45. 10.1016/s0735-1097(97)00254-4. 9316536 10.1016/s0735-1097(97)00254-4

[22] Tantrachoti P, Klomjit S, Vutthikraivit W, Prieto S, Gongora E, Nair N. Impact of preoperative atrial fibrillation in patients with left ventricular assist device: a systematic review and meta-analysis. Artif Organs. 2019;43(12):1135–43. 10.1111/aor.13523. 31250929 10.1111/aor.13523

[23] Kurihara C, Critsinelis A, Kawabori M, Sugiura T, Civitello AB, Morgan JA. Effect of preoperative atrial fibrillation on patients with chronic heart failure who undergo long-term continuous-flow LVAD implantation. ASAIO J. 2018;64(5):594–600. 10.1097/MAT.0000000000000762. 29485425 10.1097/MAT.0000000000000762

[24] Acharya D, Kazui T, Al Rameni D, Acharya T, Betterton E, Juneman E, Loyaga-Rendon R, Lotun K, Shetty R, Chatterjee A. Aortic valve disorders and left ventricular assist devices. Front Cardiovasc Med. 2023;10:1098348. 10.3389/fcvm.2023.1098348. 36910539 10.3389/fcvm.2023.1098348 PMC9996073

[25] Chung BB, Grinstein JS, Imamura T, Kruse E, Nguyen AB, Narang N, Holzhauser LH, Burkhoff D, Lang RM, Sayer GT, Uriel NY. Biventricular pacing versus right ventricular pacing in patients supported with LVAD. JACC Clin Electrophysiol. 2021;7(8):1003–9. 10.1016/j.jacep.2021.01.016. 34217657 10.1016/j.jacep.2021.01.016

[26] Usman MS, Ahmed S, Yamani N, Akhtar T, Asmi N, Siddiqi TJ, Khan SU, Doukky R, Khan MS. Meta-analysis of the effect of preoperative atrial fibrillation on outcomes after left ventricular assist device implantation. Am J Cardiol. 2019;124:158–62. 10.1016/j.amjcard.2019.03.038. 31047654 10.1016/j.amjcard.2019.03.038

[27] Stulak JM, Deo S, Schirger J, Aaronson KD, Park SJ, Joyce LD, Daly RC, Pagani FD. Preoperative atrial fibrillation increases risk of thromboembolic events after left ventricular assist device implantation. Ann Thorac Surg. 2013;96:2161–7. 10.1016/j.athoracsur.2013.07.004. 24035302 10.1016/j.athoracsur.2013.07.004

[28] Antonides CFJ, Yalcin YC, Veen KM, Muslem R, De By TMMH, Bogers AJJC, Gustafsson F, Caliskan K. Survival and adverse events in patients with atrial fibrillation at left ventricular assist device implantation: an analysis of the European registry for patients with mechanical circulatory support. Eur J Cardiothorac Surg. 2022;61(5):1164–75. 10.1093/ejcts/ezac023. 35076057 10.1093/ejcts/ezac023 PMC9070499

[29] Kirklin JK, Naftel DC, Kormos RL, Pagani FD, Myers SL, Stevenson LW, et al. Interagency registry for mechanically assisted circulatory support (INTERMACS) analysis of pump thrombosis in the heartmate II left ventricular assist device. J Heart Lung Transpl. 2014;33:12–22. 10.1016/j.healun.2013.11.001. 10.1016/j.healun.2013.11.001 24418730

[30] Estep JD, Starling RC, Horstmanshof DA, Milano CA, Selzman CH, Shah KB, ROADMAP Study Investigators, et al. Risk assessment and comparative effectiveness of left ventricular assist device and medical management in ambulatory heart failure patients: results from the ROADMAP study. J Am Coll Cardiol. 2015;66:1747–61. 10.1016/j.jacc.2015.07.075. 26483097 10.1016/j.jacc.2015.07.075

[31] Najjar SS, Slaughter MS, Pagani FD, Starling RC, McGee EC, Eckman P, et al. HVAD Bridge to transplant ADVANCE trial Investigators. An analysis of pump thrombus events in patients in the heartware ADVANCE Bridge to transplant and continued access protocol trial. J Heart Lung Transpl. 2014;33:23–34. 10.1016/j.healun.2013.12.001. 10.1016/j.healun.2013.12.001 24418731

[32] Starling RC, Moazami N, Silvestry SC, Ewald G, Rogers JG, Milano CA, et al. Unexpected abrupt increase in left ventricular assist device thrombosis. N Engl J Med. 2014;370:33–40. 10.1056/NEJMoa1313385. 24283197 10.1056/NEJMoa1313385

[33] Karuppiah S, Setty S, John R, Lupei M. Intraoperative pump thrombosis (PT) in heartmate (HM) III. J Card Surg. 2022;37:3912–5. 10.1111/jocs.16941. 36116047 10.1111/jocs.16941 PMC9826477

[34] Kewcharoen J, Shah K, Bhardwaj R, Contractor T, Turagam MK, Mandapati R, Lakkireddy D, Garg J. Surgical left atrial appendage occlusion in patients with left ventricular assist device. Pacing Clin Electrophysiol. 2022;45:567–70. 10.1111/pace.14471. 35199863 10.1111/pace.14471

[35] Sisti N, Mandoli GE, Sciaccaluga C, Valente S, Mondillo S, Cameli M. Insight into atrial fibrillation in LVAD patients: from clinical implications to prognosis. Pulse (Basel). 2020;8(1–2):2–14. 10.1159/000506600. 32999873 10.1159/000506600 PMC7506248

[36] Tsiouris A, Slaughter MS, Jeyakumar AKC, Protos AN. Left ventricular assist devices: yesterday, today, and tomorrow. J Artif Organs. 2024;27(4):335–44. 10.1007/s10047-024-01436-0. 38451441 10.1007/s10047-024-01436-0

[37] Blumer V, Ortiz Bezara M, Kittipibul V, Greene SJ, Fudim M, Hernandez GA, Chaparro S, Joyce E. Impact of atrial fibrillation on in-hospital mortality and thromboembolic complications after left ventricular assist device implantation. J Cardiovasc Transl Res. 2021;14:120–4. 10.1007/s12265-020-09968-5. 32076994 10.1007/s12265-020-09968-5

[38] Pedde D, Soltani S, Stein J, Tsyganenko D, Müller M, Schönrath F, Falk V, Potapov EV. Impact of preoperative atrial fibrillation on thromboembolic events and pump thrombosis in long-term left ventricular assist device therapy. Eur J Cardiothorac Surg. 2020;57(2):325–30. 10.1093/ejcts/ezz201. 31317177 10.1093/ejcts/ezz201

[39] Al Halabi S, Qintar M, Hussein A, Alraies MC, Jones DG, Wong T, MacDonald MR, Petrie MC, Cantillon D, Tarakji KG, Kanj M, Bhargava M, Varma N, Baranowski B, Wilkoff BL, Wazni O, Callahan T, Saliba W, Chung MK. Catheter ablation for atrial fibrillation in heart failure patients: a meta-analysis of randomized controlled trials. JACC Clin Electrophysiol. 2015;1:200–9. 10.1016/j.jacep.2015.02.018. 26258174 10.1016/j.jacep.2015.02.018 PMC4525704

[40] Prabhu S, Voskoboinik A, Kaye DM, Kistler PM. Atrial fibrillation and heart Failure - Cause or effect? Heart Lung Circ. 2017;26:967–74. 10.1016/j.hlc.2017.05.117. 28684095 10.1016/j.hlc.2017.05.117

[41] Kotecha D, Piccini JP. Atrial fibrillation in heart failure: What should we do? Eur Heart J. 2015;36:3250–7. 10.1093/eurheartj/ehv513. 26419625 10.1093/eurheartj/ehv513 PMC4670966

[42] Burashnikov A, Di Diego JM, Sicouri S, Doss MX, Sachinidis A, Barajas-Martínez H, Hu D, Minoura Y, Sydney Moise N, Kornreich BG, Chi L, Belardinelli L, Antzelevitch C. A temporal window of vulnerability for development of atrial fibrillation with advancing heart failure. Eur J Heart Fail. 2014;16:271–80. 10.1002/ejhf.28. 24464846 10.1002/ejhf.28

[43] Marrouche NF, Brachmann J, Andresen D, Siebels J, Boersma L, Jordaens L, Merkely B, Pokushalov E, Sanders P, Proff J, Schunkert H, Christ H, Vogt J. Bänsch D CASTLE-AF investigators catheter ablation for atrial fibrillation with heart failure. N Engl J Med. 2018;378:417–27. 10.1056/NEJMoa1707855. 29385358 10.1056/NEJMoa1707855

[44] Kittipibul V, Blumer V, Hernandez GA, Fudim M, Flowers R, Chaparro S, Agarwal R. Pre-operative atrial fibrillation and early right ventricular failure after left ventricular assist device implantation: a systematic review and meta-analysis. Am Heart J. 2021;239:120–8. 10.1016/j.ahj.2021.05.009. 34038705 10.1016/j.ahj.2021.05.009

[45] Oezpeker C, Zittermann A, Pühler T, Ensminger S, Gummert JF, Morshuis M. Permanent atrial fibrillation and 2 year clinical outcomes in patients with a left ventricular assist device implant. ASAIO J. 2017;63:419–24. 10.1097/MAT.0000000000000520. 28118262 10.1097/MAT.0000000000000520

[46] Hayashi H, Naka Y, Sanchez J, Takayama H, Kurlansky P, Ning Y, Topkara VK, Yuzefpolskaya M, Colombo PC, Sayer GT, Uriel N, Takeda K. Influence of atrial fibrillation on functional tricuspid regurgitation in patients with heartmate 3. J Am Heart Assoc. 2021;10:e018334. 10.1161/JAHA.120.018334. 33412902 10.1161/JAHA.120.018334 PMC7955423

[47] Hayashi H, Naka Y, Sanchez J, Takayama H, Kurlansky P, Ning Y, Topkara VK, Yuzefpolskaya M, Colombo PC, Sayer GT, Uriel N, Takeda K. Consequences of functional mitral regurgitation and atrial fibrillation in patients with left ventricular assist devices. J Heart Lung Transpl. 2020;39:1398–407. 10.1016/j.healun.2020.08.020. 10.1016/j.healun.2020.08.020 32994093

[48] Brisco MA, Sundareswaran KS, Milano CA, Feldman D, Testani JM, Ewald GA, Slaughter MS, Farrar DJ, Goldberg LR. HeartMate II clinical Investigators. Incidence, risk, and consequences of atrial arrhythmias in patients with continuous-flow left ventricular assist devices. J Card Surg. 2014;29:572–80. 10.1111/jocs.12336. 24750460 10.1111/jocs.12336

[49] Refaat M, Chemaly E, Lebeche D, Gwathmey JK, Hajjar RJ. Ventricular arrhythmias after left ventricular assist device implantation. Pacing Clin Electrophysiol. 2008;31:1246–52. 10.1111/j.1540-8159.2008.01173.x. 18811803 10.1111/j.1540-8159.2008.01173.x PMC2752870

[50] Pedrotty DM, Rame JE, Margulies KB. Management of ventricular arrhythmias in patients with ventricular assist devices. Curr Opin Cardiol. 2013;28:360–8. 10.1097/HCO.0b013e32835fb7dc. 23549232 10.1097/HCO.0b013e32835fb7dc

[51] Oz MC, Rose EA, Slater J, Kuiper JJ, Catanese KA, Levin HR. Malignant ventricular arrhythmias are well tolerated in patients receiving long-term left ventricular assist devices. J Am Coll Cardiol. 1994;24:1688–91. 7963116 10.1016/0735-1097(94)90175-9

[52] Refaat MM, Tanaka T, Kormos RL, McNamara D, Teuteberg J, Winowich S, London B, Simon MA. Survival benefit of implantable cardioverter-defibrillators in left ventricular assist device-supported heart failure patients. J Card Fail. 2012;18:140–5. 10.1016/j.cardfail.2011.10.020. 22300782 10.1016/j.cardfail.2011.10.020 PMC3272629

[53] Garan AR, Yuzefpolskaya M, Colombo PC, Morrow JP, Te-Frey R, Dano D, Takayama H, Naka Y, Garan H, Jorde UP, Uriel N. Ventricular arrhythmias and implantable cardioverter-defibrillator therapy in patients with continuous-flow left ventricular assist devices: Need for primary prevention? J Am Coll Cardiol. 2013;61:2542–50. 10.1016/j.jacc.2013.04.020. 23643502 10.1016/j.jacc.2013.04.020

[54] Enriquez AD, Calenda B, Miller MA, Anyanwu AC, Pinney SP. The role of implantable cardioverter-defibrillators in patients with continuous flow left ventricular assist devices. Circ Arrhythm Electrophysiol. 2013;6:668 –74. 10.1161/CIRCEP.113.000457. Erratum in: Circ Arrhythm Electrophysiol. 2014;7:185. 23873139 10.1161/CIRCEP.113.000457

[55] Lee W, Tay A, Subbiah RN, Walker BD, Kuchar DL, Muthiah K, Macdonald PS, Keogh AM, Kotlyar E, Jabbour A, Spratt P, Jansz PC, Granger E, Dhital K, Hayward CS. Impact of implantable cardioverter defibrillators on survival of patients with centrifugal left ventricular assist devices. Pacing Clin Electrophysiol. 2015;38:925–33. 10.1111/pace.12654. 25940215 10.1111/pace.12654

[56] Younes A, Al-Kindi SG, Alajaji W, Mackall JA, Oliveira GH. Presence of implantable cardioverter-defibrillators and wait-list mortality of patients supported with left ventricular assist devices as Bridge to heart transplantation. Int J Cardiol. 2017;231:211–5. 10.1016/j.ijcard.2016.12.148. 28043679 10.1016/j.ijcard.2016.12.148

[57] Agrawal S, Garg L, Nanda S, Sharma A, Bhatia N, Manda Y, Singh A, Fegley M, Shirani J. The role of implantable cardioverter-defibrillators in patients with continuous flow left ventricular assist devices - a meta-analysis. Int J Cardiol. 2016;222:379–84. 10.1016/j.ijcard.2016.07.257. 27505320 10.1016/j.ijcard.2016.07.257

[58] Gopinathannair R, Roukoz H, Bhan A, Ravichandran A, Ahmed MM, Familtsev D, Bhat G, Cowger J, Abdullah M, Sandesara C, Dhawan R, Birks EJ, Trivedi JR, Slaughter MS. Cardiac resynchronization therapy and clinical outcomes in continuous flow left ventricular assist device recipients. J Am Heart Assoc. 2018;7(12):e009091. 10.1161/JAHA.118.009091. 29907652 10.1161/JAHA.118.009091 PMC6220540

[59] Zook S, Ojukwu O, Khan SU, Minhas AMK, Lamba HK, Ingram KE, Kassi M. Sex-based differences in patients with left ventricular-assisted devices and strokes. JACC Adv. 2024;3(2):100817. 10.1016/j.jacadv.2023.100817. 38939388 10.1016/j.jacadv.2023.100817 PMC11198329

[60] Ko D, Rahman F, Schnabel RB, Yin X, Benjamin EJ, Christophersen IE. Atrial fibrillation in women: epidemiology, pathophysiology, presentation, and prognosis. Nat Rev Cardiol. 2016;13:321–32. 10.1038/nrcardio.2016.45. 27053455 10.1038/nrcardio.2016.45 PMC5579870

[61] Schnabel RB, Benjamin EJ. Sex and stroke risk in atrial fibrillation: more work to be done. JACC Clin Electrophysiol. 2018;4:615–7. 10.1016/j.jacep.2018.03.002. 29798788 10.1016/j.jacep.2018.03.002 PMC7657644

[62] Abrahamyan L, Sahakyan Y, Wijeysundera HC, Krahn M, Rac VE. Gender differences in utilization of specialized heart failure clinics. J Womens Health (Larchmt). 2018;27:623–9. 29319404 10.1089/jwh.2017.6461

[63] Houde S, Feldman DE, Pilote L, Beck EJ, Giannetti N, Frenette M, Ducharme A. Are there sex-related differences in specialized, multidisciplinary congestive heart failure clinics? Can J Cardiol. 2007;23:451–5. 10.1016/s0828-282x(07)70783-3. 17487289 10.1016/s0828-282x(07)70783-3 PMC2650664

[64] Lainščak M, Milinković I, Polovina M, Crespo-Leiro MG, Lund LH, Anker SD, Laroche C, Ferrari R, Coats AJS, McDonagh T, Filippatos G, Maggioni AP, Piepoli MF, Rosano GMC, Ruschitzka F, Simić D, Ašanin M, Eicher JC, Yilmaz MB, Seferović PM, European Society of Cardiology Heart Failure Long-Term Registry Investigators Group. Sex- and age-related differences in the management and outcomes of chronic heart failure: an analysis of patients from the ESC HFA EORP heart failure Long-Term registry. Eur J Heart Fail. 2020;22(1):92–102. 10.1002/ejhf.1645. 31863522 10.1002/ejhf.1645

[65] Lund LH, Carrero JJ, Farahmand B, Henriksson KM, Jonsson A, Jernberg T, Dahlstrom U. Association between enrolment in a heart failure quality registry and subsequent mortality-a nationwide cohort study. Eur J Heart Fail. 2017;19:1107–16. 28229520 10.1002/ejhf.762

[66] Savarese G, Amario D. Sex differences in heart failure. Adv Exp Med Biol. 2018;1065:529–44. 30051405 10.1007/978-3-319-77932-4_32

[67] Magnussen C, Niiranen TJ, Ojeda FM, Gianfagna F, Blankenberg S, Vartiainen E, Sans S, Pasterkamp G, Hughes M, Costanzo S, Donati MB, Jousilahti P, Linneberg A, Palosaari T, de Gaetano G, Bobak M, den Ruijter HM, Jørgensen T, Söderberg S, Kuulasmaa K, Zeller T, Iacoviello L, Salomaa V, Schnabel RB. BiomarCaRE consortium. Sex-Specific epidemiology of heart failure risk and mortality in europe: results from the biomarcare consortium. JACC Heart Fail. 2019;7:204–13. 10.1016/j.jchf.2018.08.008. 30819375 10.1016/j.jchf.2018.08.008

[68] Harmon DM, Sehrawat O, Maanja M, Wight J, Noseworthy P. Artificial intelligence for the detection and treatment of atrial fibrillation. Arrhythmia Electrophysiol Rev. 2023;12:e12. 10.15420/aer.2022.31. 10.15420/aer.2022.31 PMC10326669 37427304

[69] Attia ZI, Noseworthy PA, Lopez-Jimenez F, Asirvatham SJ, Deshmukh AJ, Gersh BJ, Carter RE, Yao X, Rabinstein AA, Erickson BJ, Kapa S, Friedman PA. An artificial intelligence-enabled ECG algorithm for the identification of patients with atrial fibrillation during sinus rhythm: a retrospective analysis of outcome prediction. Lancet. 2019;394(10201):861–7. 10.1016/S0140-6736(19)31721-0. 31378392 10.1016/S0140-6736(19)31721-0

[70] Pereira T, Tran N, Gadhoumi K, Pelter MM, Do DH, Lee RJ, Colorado R, Meisel K, Hu X. Photoplethysmography based atrial fibrillation detection: a review. NPJ Digit Med. 2020;3:3. 10.1038/s41746-019-0207-9. 31934647 10.1038/s41746-019-0207-9 PMC6954115

[71] Christopoulos G, Graff-Radford J, Lopez CL, Yao X, Attia ZI, Rabinstein AA, Petersen RC, Knopman DS, Mielke MM, Kremers W, Vemuri P, Siontis KC, Friedman PA, Noseworthy PA. Artificial intelligence-electrocardiography to predict incident atrial fibrillation: a population-based study. Circ Arrhythm Electrophysiol. 2020;13:e009355. 10.1161/CIRCEP.120.009355. 33185118 10.1161/CIRCEP.120.009355 PMC8127001

[72] Khurshid S, Friedman S, Reeder C, Di Achille P, Diamant N, Singh P, Harrington LX, Wang X, Al-Alusi MA, Sarma G, Foulkes AS, Ellinor PT, Anderson CD, Ho JE, Philippakis AA, Batra P, Lubitz SA. ECG-Based deep learning and clinical risk factors to predict atrial fibrillation. Circulation. 2022;145:122–33. 10.1161/CIRCULATIONAHA.121.057480. 34743566 10.1161/CIRCULATIONAHA.121.057480 PMC8748400

[73] Sattar Y, Song D, Sarvepalli D, Zaidi SR, Ullah W, Arshad J, Mir T, Zghouzi M, Elgendy IY, Qureshi W, Chalfoun N, Alraies M. Accuracy of pulsatile photoplethysmography applications or handheld devices vs. 12-lead ECG for atrial fibrillation screening: a systematic review and meta-analysis. J Interv Card Electrophysiol. 2022;65:33–44. 10.1007/s10840-021-01068-x. 34775555 10.1007/s10840-021-01068-x

[74] Freyer L, von Stülpnagel L, Spielbichler P, Sappler N, Wenner F, Schreinlechner M, Krasniqi A, Behroz A, Eiffener E, Zens M, Dolejsi T, Massberg S, Rizas KD, Bauer A. Rationale and design of a digital trial using smartphones to detect subclinical atrial fibrillation in a population at risk: the eHealth-based Bavarian alternative detection of atrial fibrillation (eBRAVE-AF) trial. Am Heart J. 2021;241:26–34. 10.1016/j.ahj.2021.06.008. 34252387 10.1016/j.ahj.2021.06.008

[75] Nuñez-Garcia JC, Sánchez-Puente A, Sampedro-Gómez J, Vicente-Palacios V, Jiménez-Navarro M, Oterino-Manzanas A, Jiménez-Candil J, Dorado-Diaz PI, Sánchez PL. Outcome analysis in elective electrical cardioversion of atrial fibrillation patients: development and validation of a machine learning prognostic model. J Clin Med. 2022;11:2636. 10.3390/jcm11092636. 35566761 10.3390/jcm11092636 PMC9101912

[76] Vitali F, Serenelli M, Airaksinen J, Pavasini R, Tomaszuk-Kazberuk A, Mlodawska E, Jaakkola S, Balla C, Falsetti L, Tarquinio N, Ferrari R, Squeri A, Campo G, Bertini M. CHA2DS2-VASc score predicts atrial fibrillation recurrence after cardioversion: systematic review and individual patient pooled meta-analysis. Clin Cardiol. 2019;42:358–64. 10.1002/clc.23147. 30597581 10.1002/clc.23147 PMC6712331

[77] Emren SV, Kocabaş U, Duygu H, Levent F, Şimşek EÇ, Yapan Emren Z, Tülüce S. The role of HATCH score in predicting the success rate of sinus rhythm following electrical cardioversion of atrial fibrillation. Kardiol Pol. 2016;74:978–84. 10.5603/KP.a2016.0044. 27040017 10.5603/KP.a2016.0044

[78] Rodrigo M, Alhusseini MI, Rogers AJ, Krittanawong C, Thakur S, Feng R, Ganesan P, Narayan SM. Atrial fibrillation signatures on intracardiac electrograms identified by deep learning. Comput Biol Med 202;145:105451. 10.1016/j.compbiomed.2022.105451 10.1016/j.compbiomed.2022.105451 PMC9951584 35429831

[79] Lebert J, Ravi N, Fenton FH, Christoph J. Rotor localization and phase mapping of cardiac excitation waves using deep neural networks. Front Physiol. 2021;12:782176. 10.3389/fphys.2021.782176. 34975536 10.3389/fphys.2021.782176 PMC8718715

[80] Liao S, Ragot D, Nayyar S, Suszko A, Zhang Z, Wang B, Chauhan VS. Deep learning classification of unipolar electrograms in human atrial fibrillation: application in focal source mapping. Front Physiol. 2021;12:704122. 10.3389/fphys.2021.704122. 34393823 10.3389/fphys.2021.704122 PMC8360838

[81] Tang S, Razeghi O, Kapoor R, Alhusseini MI, Fazal M, Rogers AJ, Rodrigo Bort M, Clopton P, Wang PJ, Rubin DL, Narayan SM, Baykaner T. Machine learning-enabled multimodal fusion of intra-atrial and body surface signals in prediction of atrial fibrillation ablation outcomes. Circ Arrhythm Electrophysiol. 2022;15:e010850. 10.1161/CIRCEP.122.010850. 35867397 10.1161/CIRCEP.122.010850 PMC9972736

[82] Hui J, Mauermann WJ, Stulak JM, Hanson AC, Maltais S, Barbara DW. Intensive care unit readmission after left ventricular assist device implantation: Causes, associated Factors, and association with patient mortality. Anesth Analg. 2019;128(6):1168–74. 10.1213/ANE.0000000000003847. 31094784 10.1213/ANE.0000000000003847

